# 4 pedicle screw mono-segment versus 6 pedicle screw short-segment fixation in the treatment of thoracolumbar endplate fractures: finite element analysis and clinical follow-up

**DOI:** 10.3389/fbioe.2025.1680765

**Published:** 2025-12-09

**Authors:** Kai He, Shengxiang Liu, Feng Li, Xuejun Yang, Wenhua Xing

**Affiliations:** 1 Graduate School, Inner Mongolia Medical University, Hohhot, Inner Mongolia, China; 2 Spinal Surgery Center, The Second Affiliated Hospital of Inner Mongolia Medical University, Inner Mongolia, China

**Keywords:** finite element analysis (FEA), clinical outcomes, retrospective study, mesh convergence, finite element mesh

## Abstract

**Objective:**

To compare the finite element analysis and clinical follow-up of posterior 4 pedicle screw mono-segment and 6 pedicle screw short-segment pedicle screw fixation techniques in the treatment of thoracolumbar endplate fractures of the spine.

**Methods:**

The finite element method was used to analyze the treatment of thoracolumbar upper endplate or lower endplate burst fractures with posterior 4 pedicle screw mono-segment and 6 pedicle screw short-segment pedicle screw fixation techniques (UM: upper endplate fracture + mono-segment pedicle screw fixation [UEPF + MPSF], US: upper endplate fracture + short-segment pedicle screw fixation [UEPF + SPSF], LM: lower endplate fracture + mono-segment pedicle screw fixation [LEPF + MPSF], LS: lower endplate fracture + short-segment pedicle screw fixation [LEPF + SPSF]). 2. A retrospective analysis was conducted on 77 patients with mild to moderate thoracolumbar spine fractures of type A3.1 admitted from October 2019 to October 2024. Among them, 38 patients underwent posterior 4 pedicle screw mono-segment, and 39 patients underwent posterior 6 pedicle screw short-segment fixation. The perioperative performance, clinical functional performance, and imaging performance were compared between the two groups.

**Results:**

Finite element analysis and prediction based on specific models: In fractures of the same type, the range of motion (ROM) in all directions of 4 pedicle screw mono-segment fixation showed a trend of higher values compared with 6 pedicle screw short-segment fixation, while the von Mises stress of adjacent intervertebral discs and adjacent facet joints showed a trend of lower values compared with 6-screw short-segment fixation. The 6 pedicle screw short-segment fixation model predicted that the maximum displacement of the fixed segment, the mobility of the fixed segment, and the mobility of the injured vertebra were smaller than those of 4 pedicle screw mono-segment fixation. The peak von Mises stress values of screws in the US, UM, LS, and LM groups were 386.61 Mpa, 397.60 Mpa, 302.63 Mpa, and 305.59 Mpa, respectively; the peak von Mises stress values of rods in these groups were 416.22 Mpa, 446.18 Mpa, 329.03 Mpa, and 347.47 Mpa, respectively. The stress of the injured vertebra in 6 pedicle screw short-segment fixation showed a trend of lower values compared with 4 pedicle screw mono-segment fixation. With the same fixation method, the predicted ROM of the lower endplate fracture model was larger than that of the upper endplate fracture model. The upper endplate fracture model predicted that the peak von Mises stress of adjacent intervertebral discs and facet joints appeared at the proximal end, and the stress of proximal screws was high; in contrast, the lower endplate fracture model predicted that the peak von Mises stress of adjacent intervertebral discs and facet joints appeared at the distal end, and the stress of distal screws was high. The maximum displacement of the fixed segment, the mobility of the fixed segment, and the mobility of the injured vertebra in lower endplate fractures showed a trend of lower values compared with upper endplate fractures. The stress of the screw-rod system and the injured vertebra in lower endplate fractures showed a trend of lower values compared with upper endplate fractures. 2. Clinical outcomes mainly for upper endplate fractures: ① Perioperative performance: The operation time, blood loss volume, drainage volume, time to weight-bearing time, and length of hospital stay in the 4 pedicle screw mono-segment group were all significantly lower than those in the 6 pedicle screw short-segment fixation group (*P <* 0.05). ② Clinical functional performance: Immediately after surgery, the Visual Analogue Scale (VAS) and Oswestry Disability Index (ODI) in the 4 pedicle screw mono-segment group were significantly lower than those in the 6 pedicle screw group (*P <* 0.05); there were no significant differences in VAS, ODI, or Japanese Orthopaedic Association (JOA) between the two groups at other time points (*P >* 0.05). All three scores showed significant improvement over time (*P <* 0.05). ③ Imaging performance: There were no significant differences in the anterior vertebral height (AVBH), posterior vertebral heigh (PVBH), or Cobb angle between the two groups before surgery, immediately after surgery, or at long-term follow-up (*P >* 0.05). Within each group, the AVBH, PVBH, and Cobb angle immediately after surgery and at long-term follow-up were significantly better than those before surgery (*P <* 0.05); compared with immediately after surgery, the corrected values of these indicators were lost at long-term follow-up (*P <* 0.05).

**Conclusion:**

Finite element analysis and prediction based on specific models: Both 4 pedicle screw mono-segment fixation and 6 pedicle screw short-segment fixation are effective methods for treating thoracolumbar burst fractures of the spine. The 4 pedicle screw mono-segment fixation may be suitable for patients with normal bone mineral density and mild-to-moderate fractures, with advantages of preserving spinal mobility and reducing the risk of adjacent segment degeneration. The 6 pedicle screw short-segment fixation has a wider application range, and its advantages lie in better stability and stress dispersion. For upper endplate fractures, the adjacent segment at the proximal end is the stress concentration area, and the proximal screws bear the maximum stress; in contrast, for lower endplate fractures, the adjacent segment at the distal end is the stress concentration area, and the distal screws bear the maximum stress. The stability gap between 4 pedicle screw mono-segment fixation and 6 pedicle screw short-segment fixation is smaller in lower endplate fractures than in upper endplate fractures. Compared with upper endplate fractures, lower endplate fractures show better overall performance and may have a better prognosis. However, different finite element models may be required in the future to reduce the impact of individual differences. 2. Clinical outcomes mainly for upper endplate fractures: For mild-to-moderate fractures, the 4 pedicle screw mono-segment fixation achieves the same reduction effect as the 6 pedicle screw short-segment fixation both immediately after surgery and during long-term internal fixation removal. Moreover, it has advantages such as shorter operation time, smaller incision, less blood loss, earlier weight-bearing time, shorter hospital stay, less early postoperative pain, and faster functional recovery. However, more cases of lower endplate fractures may need to be collected in the future for further verification.

## 1 Introduction

Spinal fracture refers to the disruption of the continuity and integrity of the spinal bones. The thoracolumbar segment (T10-L2) is located in the transition zone between the less flexible thoracic spine and the highly flexible lumbar spine; it is also the junction of the physiological kyphosis of the thoracic spine and the physiological lordosis of the lumbar spine. Additionally, this segment is connected to the relatively fixed thoracic cage, which limits its mobility. These multiple factors lead to stress concentration in the thoracolumbar segment, making it the most common site for spinal fractures, accounting for approximately 90% of all spinal fractures. Among these, 10%–20% are burst fractures (Dai et al., 2007; Murphy et al., 2020; Spiegl et al., 2021; Wood et al., 2005). Conservative treatment methods include bed rest immobilization, brace therapy supplemented with analgesic drugs; however, delayed conservative treatment may result in the deterioration of neurological function in 17% of patients (Charles and Steib, 2015; Tan et al., 2022). The purpose of the surgery is to relieve severe pain, decompress the spinal cord and nerves, restore spinal stability, and correct kyphotic deformity (Li et al., 2021). However, there is controversy regarding the guidelines for surgical approaches. First, regarding whether to place screws in the injured vertebra, multiple pieces of current clinical evidence indicate that screw placement through the injured vertebra helps improve biomechanical stability. Only when the pedicle is damaged and no longer suitable for screw placement is screw fixation spanning the injured vertebra considered (Anekstein et al., 2007; B et al., 2011; Guven et al., 2009; Mahar et al., 2007). Second, regarding the number of screws used, this mainly depends on the severity of spinal injury. The commonly used numbers are 4 screws and 6 screws; only in cases of three-column fractures or osteoporotic vertebral compression fractures is long-segment fixation with more screws adopted to enhance stability (Zhang et al., 2024). Finally, whether open decompression is needed mainly depends on the size of the bone fragment that has entered the spinal canal. Generally, if the bone fragment occupies less than 50% of the spinal canal volume, open decompression is not necessary; decompression can be achieved by pushing the fragmented bone into the spinal canal through the process of straightening the folded posterior longitudinal ligament. (Lamichhane et al., 2025; Song et al., 2022). With the current development and advancement of medical standards, while pursuing treatment efficacy, we are paying increasing attention to the concept of enhanced recovery after surgery (ERAS). On the premise of restoring vertebral height and correcting kyphotic deformity, posterior 4 pedicle screw mono-segment fixation has advantages such as lower surgical difficulty, minimal trauma, reduced risks, and fewer complications. However, relevant research on this technique is currently lacking (Wang et al., 2012).

This study conducted a comparative analysis of 4 pedicle screw mono-segment fixation and 6 pedicle screw short-segment fixation for thoracolumbar burst fractures. Due to the anatomical asymmetry of the superior and inferior structures of the vertebra—specifically, the pedicles are located at the level of the upper endplate and far from the lower endplate, and screws are inserted into the vertebral body through the pedicles—screws may be positioned in the fractured area when an upper endplate fracture occurs, whereas screws are almost entirely placed in intact vertebral structures when a lower endplate fracture occurs. To investigate whether there are differences between upper and lower endplate fractures, this study further subdivided thoracolumbar burst fractures into type A3.1 upper endplate fractures and type A3.1 lower endplate fractures for separate analysis. Ultimately, this study analyzed and compared four groups of research subjects, namely: US, UM, LS, LM.

## 2 Materials and methods

### 2.1 Finite element analysis

#### 2.1.1 Establishment of the normal model

A healthy 35-year-old male volunteer signed the informed consent form. This study was approved by the ethics committee (Approval No.: EFY20250094).

Geometric details of the T10-L2 vertebrae from the healthy young male were obtained using a 64-slice spiral CT scanner (GE Healthcare, USA). Spinal images from the CT scan were imported into Mimics Software 20.0 (Materialise, Belgium) in DICOM format to extract three-dimensional (3D) contour tissue outlines. These outlines were then imported as STL files into Geomagic 12 (Geomagic, USA) for reverse engineering reconstruction. The extracted 3D contours were solidified to generate 3D models in IGES file format. Based on the above model, SolidWorks 2015 (Dassault Systèmes, France) was used to construct the endplates, annulus fibrosus, nucleus pulposus, cortical bone, cancellous bone, and articular cartilage. The volume ratio of the annulus fibrosus to the nucleus pulposus was set to 6:4, and the nucleus pulposus was defined as a fluid-impermeable material. The intervertebral disc and vertebral body were set as bonded contacts, while the facet joint surfaces were set as surface-to-surface contacts with a friction coefficient of 0.1. The model was then imported into ANSYS Workbench 18.0 (ANSYS, USA) to construct various ligaments, including the anterior longitudinal ligament, posterior longitudinal ligament, ligamentum flavum, supraspinous ligament, interspinous ligament, intertransverse ligament, and capsular ligament. The node-element counts for each group were as follows: US (326,387–1,008,902), UM (294,629–886,300), LS (324,117–1,000,149), and LM (291,995–877,833). The assigned material properties are presented in Table 1.

**TABLE 1:** A represents the material parameters of the finite element; B represents the mesh convergence test.

Component	Elastic modulus (Mpa)	Poisson’s ratio	Cross section (mm²)	Element type
Cortical bone	12000	0.3		Solid187
Spongy bone	100	0.3		Solid187
End plate	1,000	0.4		Solid186
Posterior structure	3,500	0.25		Solid187
Zygapophyseal joint	10	0.4		Solid186
Nucleus pulposus	0.2	0.49		Solid186
Annulus fibrosus	4.2	0.45		Solid186
Anterior longitudinal ligament	20	0.3	63.7	Link180
Posterior longitudinal ligament	20	0.3	20	Link180
Intertransverse ligament	58.7	0.3	3.6	Link180
Ligamentum flavum	19.5	0.3	40	Link180
Interspinal ligament	11.6	0.3	40	Link180
Supraspinous ligament	15	0.3	30	Link180
Capsular ligament of the joint	32.9	0.3	60	Link180
Pedicle screws and rods	110000	0.3		Solid187

#### 2.1.2 Establishment of the fracture model

**FIGURE 1:**
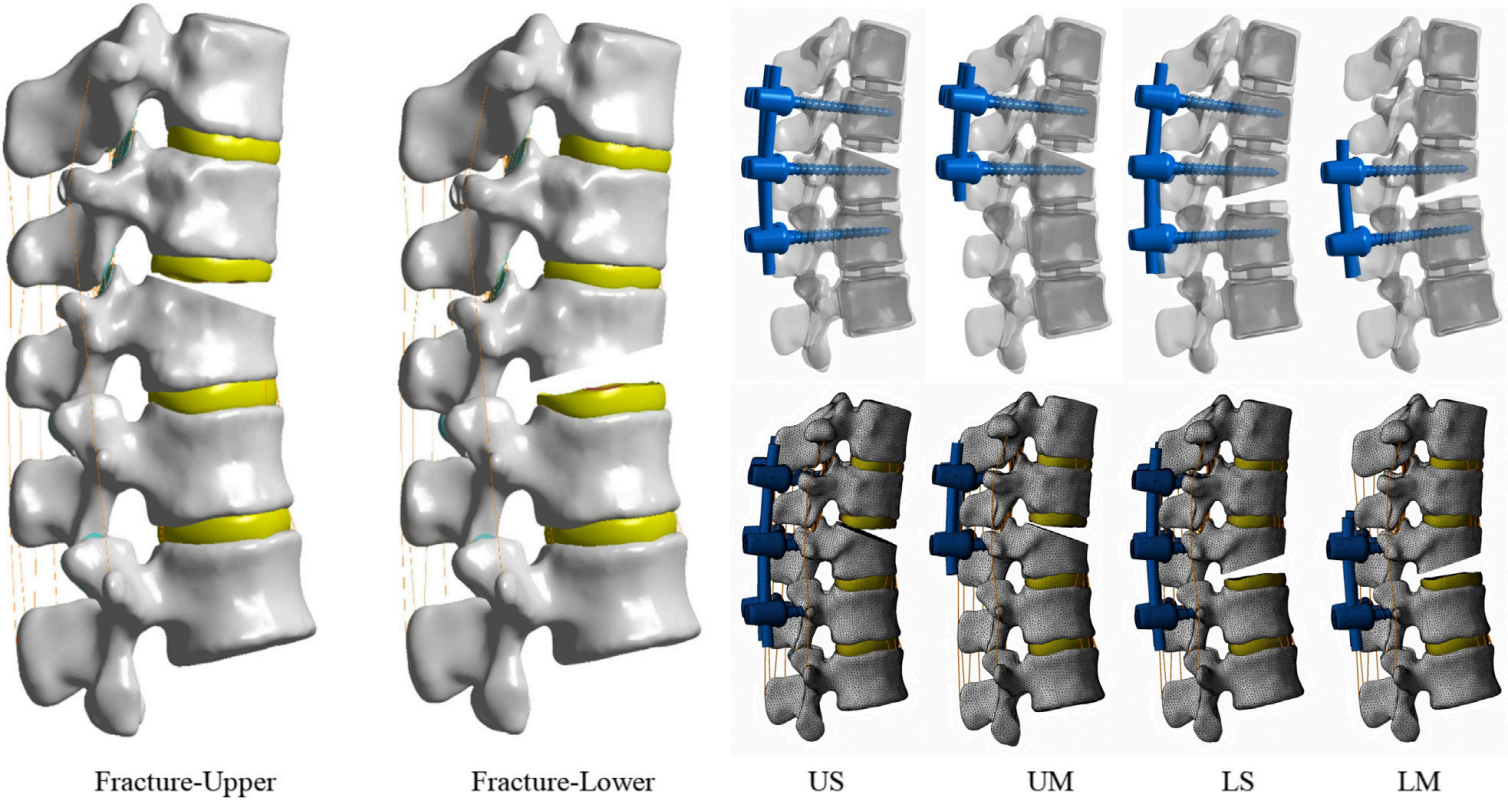
Two types of fracture models, including upper endplate fracture and lower endplate fracture. Four types of internal fixation models, including US, UM, LS, and LM.

In finite element analysis, the “V-shaped” osteotomy is commonly adopted for the establishment of fracture models (Basaran et al., 2019). Since no previous studies have explored endplate fractures to date, this study constructed the model by combining the V-shaped osteotomy method with the location and scope of endplate fractures. Endplate fractures are located on one side of the vertebral body, and their scope usually accounts for 1/3 to 1/2 of the height of the anterior edge of the vertebral body. Therefore, in this model, SolidWorks software was used to perform “V-shaped” osteotomy on approximately 1/3 of the height of the anterior edge of the upper or lower part of the vertebral body to simulate upper endplate fractures and lower endplate fractures (Figure 1).

#### 2.1.3 Establishment of fixation models

Mono-segment fixation or Short-segment fixation was performed for thoracolumbar upper endplate fractures and lower endplate fractures, and four types of fixation models were established: the UM model, US model, LM model, and LS model (Figure 1). Among them, UM stands for mono-segment fixation for upper endplate fractures, US stands for short-segment fixation for upper endplate fractures, LM stands for mono-segment fixation for lower endplate fractures, and LS stands for short-segment fixation for lower endplate fractures. In clinical practice, most patients with thoracolumbar fractures use 6–6.5 mm screws. The selection criterion for screws is that the screw diameter should be 1–2 mm smaller than the “minimum inner diameter” of the pedicle, following the principle of “the thicker the better” (Öztürk et al., 2024). For this volunteer, the inner diameter of the pedicle isthmus of T11–L1 measured by CT ranges from 7 to 7.5 mm; therefore, 6 mm screws were used. Using the modeling function of SolidWorks, modeling was performed based on the dimensions of the screw-rod system (screw diameter: 6.0 mm, total screw length: 40 mm, rod diameter: 5.5 mm, rod length: the same as the fixed segment). Subsequently, the assembly function of SolidWorks was used to combine the screw-rod system first, followed by precise integration with the vertebral body. In the model, screws were meticulously simulated, including the intermediate polyaxial screws used in 6 pedicle screw fixation (all other screws were fixed screws) and screw threads. Among these settings: the contact between the polyaxial screw head and the screw shaft was defined as “no separation” to allow sliding; the contact between the screw threads and the vertebral body was set as “bonded contact” to prevent sliding—all screw threads and the vertebral body were treated as an integrated unit, and the threads had no impact on stress changes. The element types, material properties, and ligament cross-sectional areas are presented in Table 1.

#### 2.1.4 Mesh convergence test

Since this study mainly focuses on the safety of internal fixation screws in different combinations under various fracture patterns, the maximum equivalent stress of the internal fixation screws under different mesh sizes was compared by adjusting the mesh size of the internal fixation screws. The mesh sizes were set to 0.5 mm, 1.0 mm, 2.0 mm, 3.0 mm, and 5.0 mm, respectively. The maximum equivalent stress of the screws was selected as the observation index, and the relationship between the stress and mesh size is shown in Table 1.

When the mesh size is less than 1.0 mm, the result differs by 2.1% from that when the mesh size is 0.5 mm. Considering computational efficiency, a mesh size of 1.0 mm was selected as the mesh dimension for subsequent finite element model studies.

#### 2.1.5 Boundary conditions and loads

The inferior edge of the L2 vertebral body was fixed to restrict the movement of the L2 lower endplate in all directions. Referring to the standing posture and weight-bearing status of the volunteer (height: 170 cm, weight: 65 kg) during the rehabilitation period, a vertical load of 400 N was applied to the upper surface of the T10 vertebral body, and a load of 7.5 Nm was applied to the upper surface of the T10 vertebral body to simulate movements including flexion, extension, left lateral bending, right lateral bending, left rotation, and right rotation. The following parameters were analyzed for all segments: ROM of the T10-L2, von Mises stress of adjacent intervertebral discs, von Mises stress of adjacent facet joints, maximum displacement of the fixed segment, ROM of the fixed segment, ROM of the injured T12 vertebral body, stress of the injured T12 vertebral body, and stress of pedicle screws and rods. Von Mises stress converts multi-directional stresses in three-dimensional space (tensile stress, compressive stress, shear stress, etc.) into an equivalent scalar value. Facet joints and intervertebral discs bear compressive, shear, bending, and even torsional stresses during physiological activities, which is also a state of “multi-directional stress superposition”—this fully aligns with the concept of “complex stress simplification” of von Mises stress. Von Mises stress can intuitively reflect the overall stress intensity of tissues through a single numerical value; therefore, in this study, von Mises stress was used to represent the stress of intervertebral discs and facet joints, and this method has also been applied in some previous studies (Wang et al., 2024).

### 2.2 Clinical follow-up analysis

#### 2.2.1 General information

A retrospective analysis was conducted on the data of 77 patients with mild thoracolumbar type A3 spinal fractures admitted from October 2021 to October 2024. The inclusion criteria were as follows: ① A load sharing classification (LSC) score of ≤6 based on X-ray and CT examinations; ② Intact posterior ligamentous complex (PLC) confirmed by MRI; ③ Bone mineral density (BMD) > −2.5; ④ American Spinal Injury Association (ASIA) neurological function grade E; ⑤ Fracture segments within the range of T11–L2. Among these patients, 38 underwent posterior 4 pedicle screw mono-segment fixation, and 39 underwent posterior 6 pedicle screw short-segment fixation. This study obtained informed consent from all patients and was approved by the ethics committee.

#### 2.2.2 Sample size calculation

The minimum sample size for this study was calculated using G*Power 3.1.9.7 software. Based on the results of the pilot experiment [preoperative anterior vertebral height: 21.78 ± 2.91 vs. 23.90 ± 3.33], the effect size (d) was set to 0.68, the significance level (α) was set to 0.05 (two-tailed), and the statistical power (1-β) was set to 0.80. For the independent samples t-test, the calculated minimum sample size was 36 cases. The final sample size was determined to be more than 36 cases per group to ensure the study had sufficient statistical test power. A total of 77 patients were ultimately included (38 cases in the single-segment fixation group and 39 cases in the short-segment fixation group), with an actual statistical power of 0.84.

#### 2.2.3 Surgical methods

After general anesthesia, the patient was placed in a prone position with slight hyperextension. The fracture site was localized under fluoroscopy, followed by disinfection and draping.

4 Pedicle Screw Mono-Segment Fixation: The injured vertebra and either the vertebra above or below it were localized (for upper endplate fractures, the injured vertebra and the vertebra above it were localized; for lower endplate fractures, the injured vertebra and the vertebra below it were localized). An incision was made sequentially through the skin, subcutaneous tissue, fascia, and muscles to expose the screw entry points. A No. 1 drill was used to create a pilot hole, which was then enlarged with a No. 2 drill. A probe was used to confirm that the wall of the channel was composed of bone. A positioning pin was inserted, and its position was verified under fluoroscopy. Once the position was satisfactory, 4 pedicle screws were implanted. A pre-bent rod was inserted, and sequential distraction and locking were performed. The reduction effect was observed under fluoroscopy; when the reduction was satisfactory, the screw caps were locked. A drainage tube was placed, followed by irrigation and suturing.

6 Pedicle Screw Short-Segment Fixation: The injured vertebra, the vertebra above it, and the vertebra below it were localized. An incision was made sequentially through the skin, subcutaneous tissue, fascia, and muscles to expose the screw entry points. A No. 1 drill was used to create a pilot hole, which was then enlarged with a No. 2 drill. A probe was used to confirm that the wall of the channel was composed of bone. A positioning pin was inserted, and its position was verified under fluoroscopy. Once the position was satisfactory, 6 pedicle screws were implanted. A pre-bent rod was inserted, and sequential distraction and locking were performed. The reduction effect was observed under fluoroscopy; when the reduction was satisfactory, the screw caps were locked. A drainage tube was placed, followed by irrigation and suturing.

Postoperatively, measures such as improving ventilation, fluid replacement, anticoagulation, pain management, and incision dressing changes were administered. The drainage tube was removed when no fluid outflow was observed. Patients were instructed on rehabilitation exercises and advised to wear a brace. Regular follow-up examinations were scheduled every 3 months postoperatively.

#### 2.2.4 Measurement tools and evaluation indicators

##### 2.2.4.1 Equipment

SRO33100 DR (Philips, Netherlands), Aquilion 64-slice spiral CT scanner (Toshiba (China) Co., Ltd. Guangzhou Branch), MAGNETOM Skyra 3.0T superconducting MRI (Siemens, Germany), and VISIONPACS clinical browsing system (with built-in measurement software).

##### 2.2.4.2 Recorded outcomes

① Perioperative parameters: Operation time, incision length, blood loss, drainage volume, weight-bearing time, hospital time. ② Clinical function: VAS, ODI, JOA. ③ Imaging parameters: AVBH, PVBH, Cobb angle. Clinical function and imaging were evaluated at three time points: pre-operative, immediately post-operatively (when the drainage tube was removed), and long-term follow-up (when internal fixation was removed).

X-ray radiological data were measured jointly by a senior spinal surgeon and a radiologist, who were blinded to all patient information. The VAS, ODI, and JOA scores were collected by the department’s research assistant, who was also blinded to patient information, before each patient’s admission and promptly after surgery.

#### 2.2.5 Statistical methods

Statistical analyses were performed using SPSS 26.0 software, and graphs were generated with GraphPad Prism 10.0 software. For categorical data, the chi-square test was applied, and data were expressed as count (percentage). For continuous data that met the assumptions of normal distribution and homogeneous variance between the two groups, the independent samples t-test was used, and data were expressed as mean ± standard deviation. For continuous data that did not meet the assumptions of normal distribution or homogeneous variance between the two groups, the Mann-Whitney U test was used, and data were expressed as median [interquartile range]. For repeated-measures data, repeated measures analysis of variance (ANOVA) was used, and the Bonferroni *post hoc* test was applied for intra-group pairwise comparisons. *P* < 0.05 was considered to indicate a statistically significant difference.

## 3 Results

### 3.1 Finite element analysis

#### 3.1.1 Validation of the validity of the intact T10-L2 model

To validate the validity of the intact T10-L2 model, a vertical compressive load of 150 N and a torque of 10 Nm were applied to the model with reference to published literature (Su et al., 2018). The range of motion (ROM) of the intact T10-L2 spinal model was measured under flexion, extension, left lateral bending, right lateral bending, left rotation, and right rotation. The ROM values were 5.28°, 4.92°, 2.43°, 2.36°, 4.52°, and 4.68°, respectively. The obtained results were compared and analyzed with the findings from studies by Su et al. (2018), Pflugmacher et al. (2004), and Li et al. (2014) (Table 2).

**TABLE 2:** Comparison between the normal finite element model in this study and the finite element models from previous studies.

States of motion	ROM of T11-L2 (°)			
	Present study	Yunshan Su’s study	Pflugmacher’s study	Changqing Li’s study
Flexion	5.28	5.9	5.3 ± 1.0	4.6 ± 0.6
Extension	4.92	4.6	5.7 ± 1.0	4.5 ± 1.1
Left axial rotation	2.43	2.6	2.1 ± 0.5	3.2 ± 0.8
Right axial rotation	2.36	2.1	2.1 ± 0.5	3.2 ± 0.6
Left lateral bending	4.52	4.5	4.3 ± 0.6	4.6 ± 0.7
Right lateral bending	4.68	5.3	4.3 ± 0.6	4.8 ± 0.5

#### 3.1.2 Range of motion (ROM) of all segments

Through finite element analysis and prediction of specific models, compared with the ROM of all segments in normal individuals, the ROM of all segments after fixation decreased. For all groups, the peak ROM of all segments occurred under the flexion condition, while the minimum ROM of all segments occurred under the rotation condition.

The ROM of all segments in the UM group was greater than that in the US group under all 6 conditions, and the ROM of all segments in the LM group was greater than that in the LS group under all 6 conditions. Under the conditions of flexion (peak ROM), extension, left lateral bending, right lateral bending, and left rotation, the ROM of all segments in the UM group was smaller than that in the LM group. Compared with the LM group, the ROM of the UM group decreased by 8.68%, 22.18%, 1.35%, 14.71%, and 19.36% respectively under the above conditions. The ROM of all segments in the US group was smaller than that in the LS group under all 6 conditions. Compared with the LS group, the ROM of the US group decreased by 5.54% (flexion), 54.42% (extension), 4.18% (left lateral bending), 20.25% (right lateral bending), 1.84% (left rotation), and 4.71% (right rotation). The ROM values are presented in Table 3 and Figure 5.

**TABLE 3:** Total ROM of the T10-L2 segments under 6 conditions (flexion, extension, left lateral bending, right lateral bending, left rotation, and right rotation) for the normal model and the four fixation methods.

Model type	ROM of T11-L2 (°)					
	Flexion	Extension	Left lateral bending	Right lateral bending	Left axial rotation	Right axial rotation
Normal model	11.975	9.824	5.185	5.207	7.864	7.903
US	7.451	1.835	1.215	1.268	1.066	1.073
UM	8.924	3.726	1.457	1.594	1.249	1.326
LS	7.888	4.026	1.268	1.59	1.086	1.126
LM	9.699	4.788	1.477	1.869	1.322	1.313

#### 3.1.3 Von mises stress of adjacent intervertebral discs

The von Mises stress of all adjacent intervertebral discs increased after fixation. For each group, the maximum von Mises stress of adjacent intervertebral discs occurred under the lateral bending condition, while the minimum von Mises stress of adjacent intervertebral discs in each group occurred under the flexion or extension condition.

The peak von Mises stress of adjacent intervertebral discs was 22.102 Mpa, which appeared in the proximal adjacent intervertebral disc of the US group under the left lateral bending condition. The average stress values of the proximal adjacent intervertebral discs in the US, UM, LS, and LM groups were 15.92 Mpa, 14.95 Mpa, 14.10 Mpa, and 12.35 Mpa, respectively. The average stress values of the distal adjacent intervertebral discs in the US, UM, LS, and LM groups were 12.42 Mpa, 8.96 Mpa, 12.62 Mpa, and 12.17 Mpa, respectively. The von Mises stress of adjacent intervertebral discs in the US group was greater than that in the UM group under all 6 conditions, and the von Mises stress of adjacent intervertebral discs in the LS group was also greater than that in the LM group under all 6 conditions.

**FIGURE 2:**
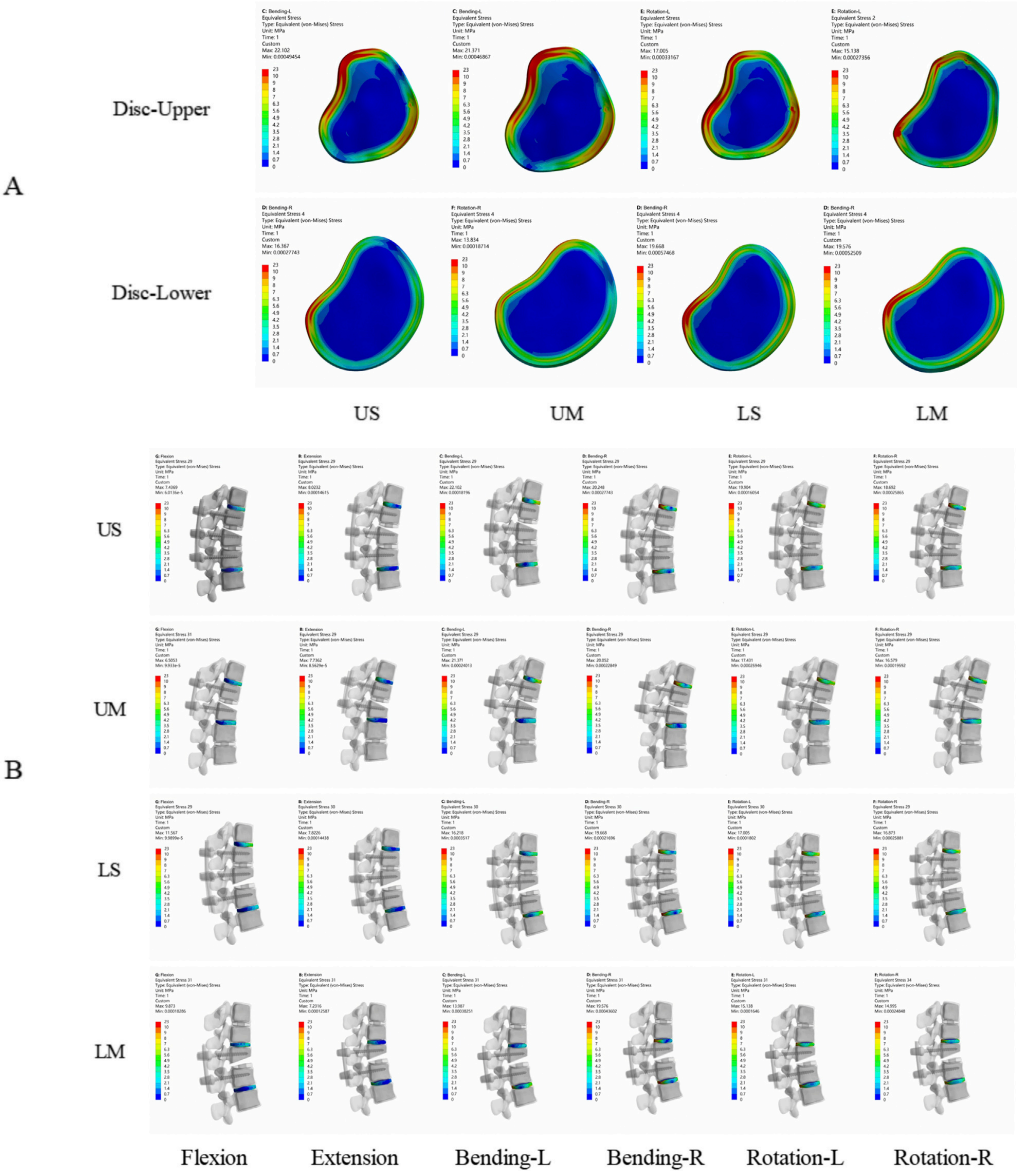
Von Mises stress nephograms of adjacent intervertebral discs in the 4 finite element models. **(A)** is the axial view, showing the peak stress of adjacent intervertebral discs at the proximal and distal ends of the 4 fixation models. **(B)** is the lateral view, showing the stress of adjacent intervertebral discs in the 4 fixation models under flexion, extension, left lateral bending, right lateral bending, left rotation, and right rotation. The stress of adjacent intervertebral discs is mainly concentrated in the anterior and posterior parts of the intervertebral discs.

**FIGURE 3:**
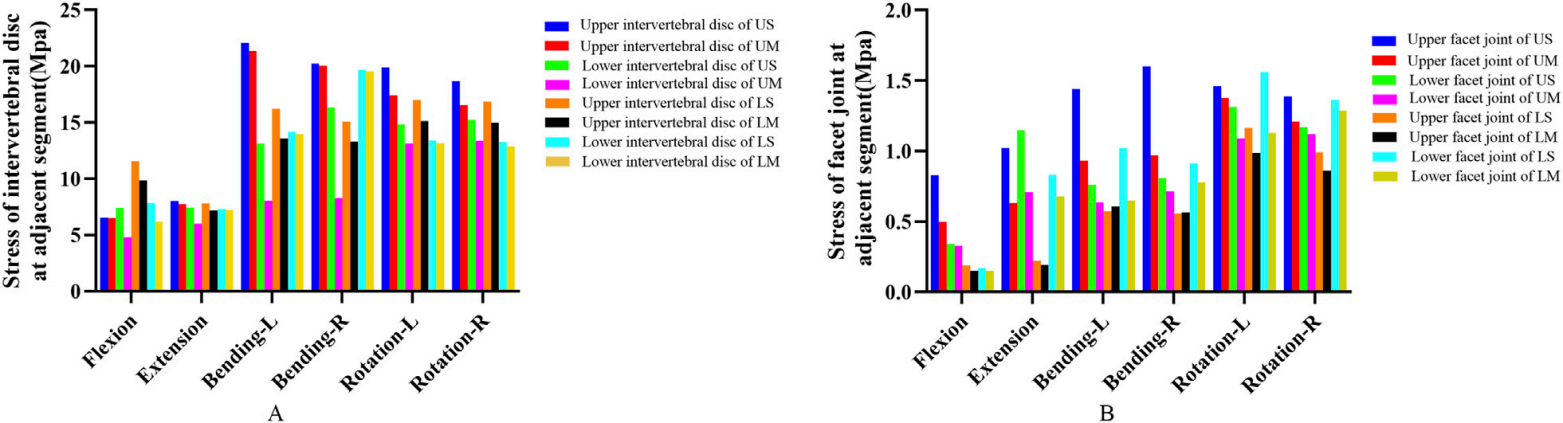
**(A)** shows the von Mises stress of adjacent intervertebral discs; **(B)** shows the von Mises stress of adjacent facet joints.

The peak von Mises stress of adjacent intervertebral discs in the UM and US groups was observed in the upper intervertebral disc, while the peak von Mises stress of adjacent intervertebral discs in the LM and LS groups was observed in the lower intervertebral disc. The peak von Mises stress of adjacent intervertebral discs in the UM group was higher than that in the LM group, and the peak von Mises stress of adjacent intervertebral discs in the US group was higher than that in the LS group. Compared with the LM group, the peak von Mises stress of adjacent intervertebral discs in the UM group increased by 9.17%. Compared with the LS group, the peak von Mises stress of adjacent intervertebral discs in the US group increased by 12.38%. For upper endplate fractures, the peak von Mises stress of adjacent intervertebral discs occurred in the proximal disc; for lower endplate fractures, the peak von Mises stress of adjacent intervertebral discs occurred in the distal disc. The von Mises stress of adjacent intervertebral discs is shown in Figures 2, 3.

#### 3.1.4 Von mises stress of adjacent facet joints

The von Mises stress of all adjacent facet joints increased after fixation. For each group, the maximum von Mises stress of adjacent facet joints occurred under rotational conditions, while the minimum von Mises stress of adjacent facet joints in each group occurred under flexion conditions.

The average von Mises stress values of the proximal adjacent facet joints in the US, UM, LS, and LM groups were 1.2909 Mpa, 0.9372 Mpa, 0.6168 Mpa, and 0.5611 Mpa, respectively. The average von Mises stress values of the distal adjacent facet joints in the US, UM, LS, and LM groups were 0.9247 Mpa, 0.7675 Mpa, 0.9776 Mpa, and 0.7795 Mpa, respectively. The von Mises stress of adjacent facet joints in the US group was greater than that in the UM group under all 6 conditions, and the von Mises stress of adjacent facet joints in the LS group was greater than that in the LM group under all 6 conditions. The peak von Mises stress of adjacent facet joints in the US and UM groups occurred in the proximal joints, while the peak von Mises stress of adjacent facet joints in the LS and LM groups occurred in the distal joints. The peak von Mises stress of adjacent facet joints in the US group was greater than that in the LS group, and the peak von Mises stress of adjacent facet joints in the UM group was greater than that in the LM group. The von Mises stress of adjacent facet joints is shown in Figure 3.

#### 3.1.5 Maximum displacement of the fixed segment

The maximum displacement of the fixed segment is defined as the maximum vertical displacement between the upper vertebral body and the posterior part of the lower vertebral body. The maximum displacement of the fixed segment decreased in all groups. For each group, the peak maximum displacement of the fixed segment occurred under the flexion condition, while the minimum maximum displacement of the fixed segment in each group occurred under the rotation condition.

The peak values of the maximum displacement of the fixed segment in the US, UM, LS, and LM groups were −9.985 mm, −11.027 mm, −7.5681 mm, and −8.7547 mm, respectively. The average values of the maximum displacement of the fixed segment in the US, UM, LS, and LM groups were −3.3395 mm, −3.9784 mm, −3.1379 mm, and −3.5410 mm, respectively. Under all 6 conditions, the maximum displacement of the fixed segment in the US group was smaller than that in the UM group, and the maximum displacement of the fixed segment in the LS group was smaller than that in the LM group.

**FIGURE 4:**
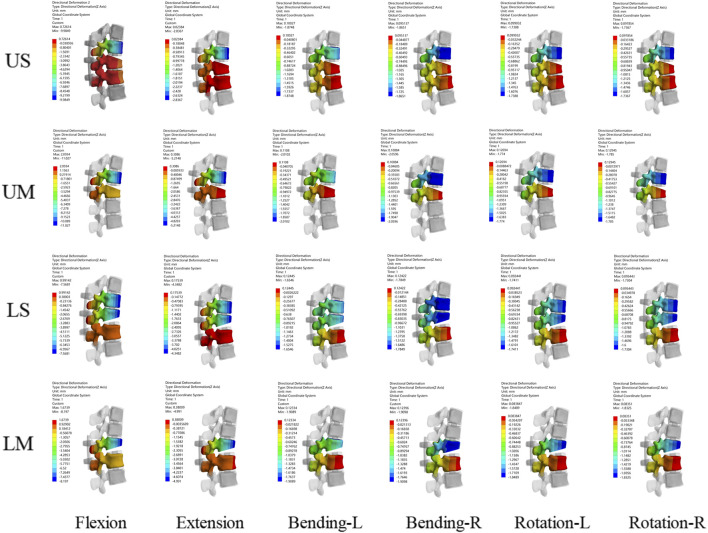
Displacement nephograms for the four fixation methods show the maximum displacement of the fixed segment under six conditions including flexion, extension, left lateral bending, right lateral bending, left rotation, and right rotation. The part with the largest displacement in the fixed segment is located in the distal vertebral body.

**FIGURE 5:**
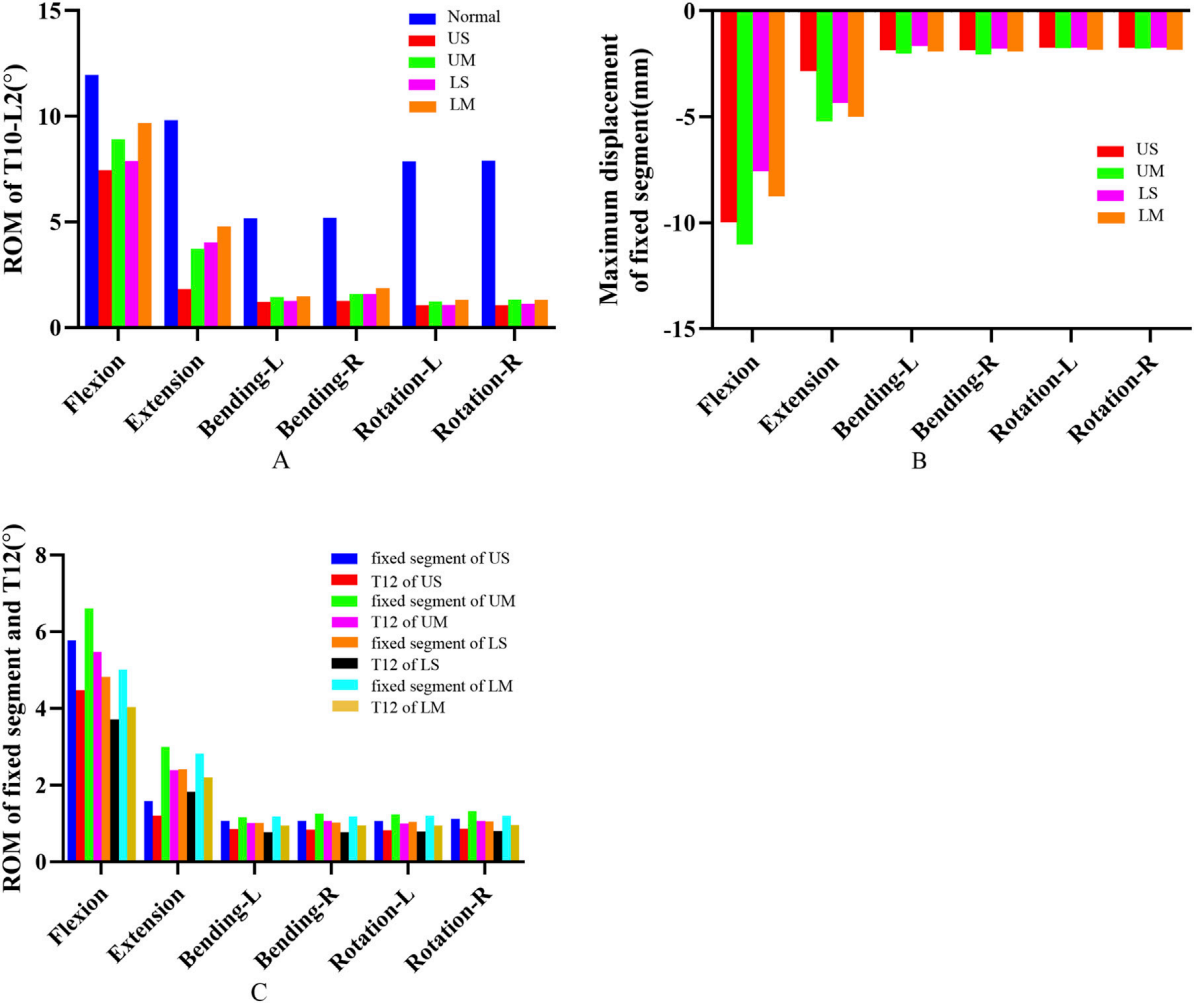
**(A)** shows the ROM of T10-L2 segments for the four fixation methods; **(B)** shows the maximum displacement of the fixed segment for the four fixation methods; **(C)** shows the ROM of the fixed segment and the injured T12 vertebra for the four fixation methods.

The peak value of the maximum displacement of the fixed segment in the UM group was greater than that in the LM group, and the peak value of the maximum displacement of the fixed segment in the US group was greater than that in the LS group. Compared with the LM group, the peak value of the maximum displacement of the fixed segment in the UM group was 20.63% larger. Compared with the LS group, the peak value of the maximum displacement of the fixed segment in the US group was 24.21% larger. The maximum displacement of the fixed segment is shown in Figures 4, 5.

#### 3.1.6 ROM of the fixed segment and the injured T12 vertebra

The ROM of all fixed segments decreased, and the maximum ROM of the fixed segments occurred under flexion. The peak ROM values of the fixed segments in the US, UM, LS, and LM groups were 5.778°, 6.608°, 4.825°, and 5.016°, respectively. Under all 6 conditions, the ROM of the fixed segment in the US group was smaller than that in the UM group, and the ROM of the fixed segment in the LS group was smaller than that in the LM group. Additionally, the peak ROM of the fixed segment in the US group was greater than that in the LS group, and the peak ROM of the fixed segment in the UM group was greater than that in the LM group.

The ROM of all injured T12 vertebrae decreased, and the ROM of the injured T12 vertebrae also reached its maximum under flexion. The peak ROM values of the injured T12 vertebrae in the US, UM, LS, and LM groups were 4.476°, 5.479°, 3.714°, and 4.038°, respectively. Under all 6 conditions, the ROM of the injured T12 vertebra in the US group was smaller than that in the UM group, and the ROM of the injured T12 vertebra in the LS group was smaller than that in the LM group. Moreover, the peak ROM of the injured T12 vertebra in the US group was greater than that in the LS group, and the peak ROM of the injured T12 vertebra in the UM group was greater than that in the LM group.

The ratio of the ROM of the injured T12 vertebra to the ROM of the fixed segment is defined as the “instability ratio”. Under all 6 conditions, the instability ratio of the US group was smaller than that of the UM group, and the instability ratio of the LS group was smaller than that of the LM group. The average instability ratio values across the 6 conditions in the US, UM, LS, and LM groups were 77.66%, 82.59%, 76.17%, and 79.33%, respectively. The average instability ratio of the UM group was 6.35% higher than that of the US group, and the average instability ratio of the LM group was 4.15% higher than that of the LS group. The maximum displacement of the fixed segment, as well as the ROM of the fixed segment and the injured T12 vertebra, are shown in Figure 5.

#### 3.1.7 Von mises stress of screw-rod system

The peak von Mises stress of screws in each group occurred under the flexion condition. The peak von Mises stress values of screws in the US, UM, LS, and LM groups were 386.61 Mpa, 397.60 Mpa, 302.63 Mpa, and 305.59 Mpa, respectively. The peak von Mises stress of rods in each group also occurred under flexion. The peak von Mises stress values of rods in the US, UM, LS, and LM groups were 416.22 Mpa, 446.18 Mpa, 329.03 Mpa, and 347.47 Mpa, respectively.

**FIGURE 6:**
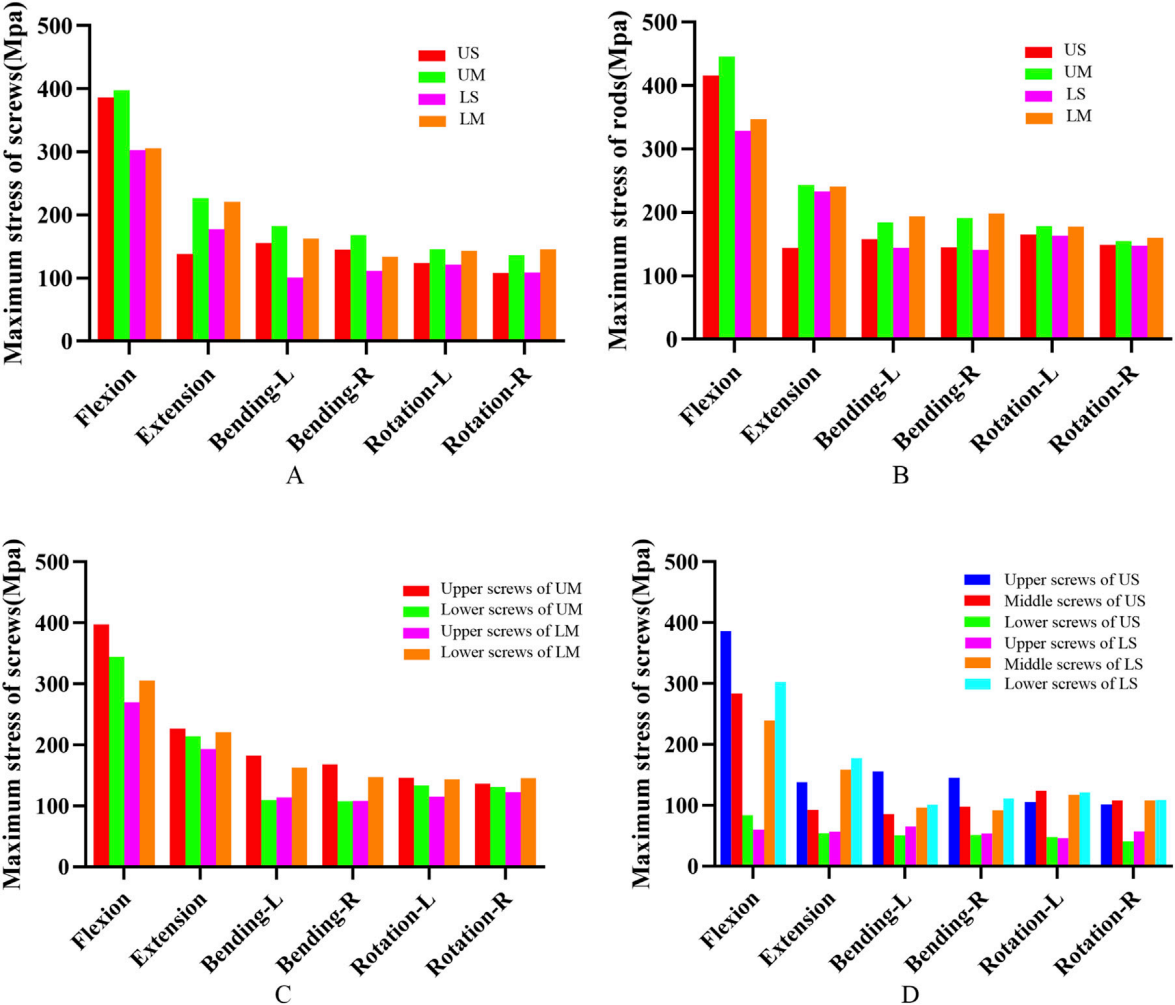
**(A)** shows the von Mises stress of pedicle screws for the four fixation methods; **(B)** shows the von Mises stress of rods for the four fixation methods; **(C)** shows the von Mises stress of screws at different positions in the UM and LM groups; **(D)** shows the von Mises stress of screws at different positions in the US and LS groups.

**FIGURE 7:**
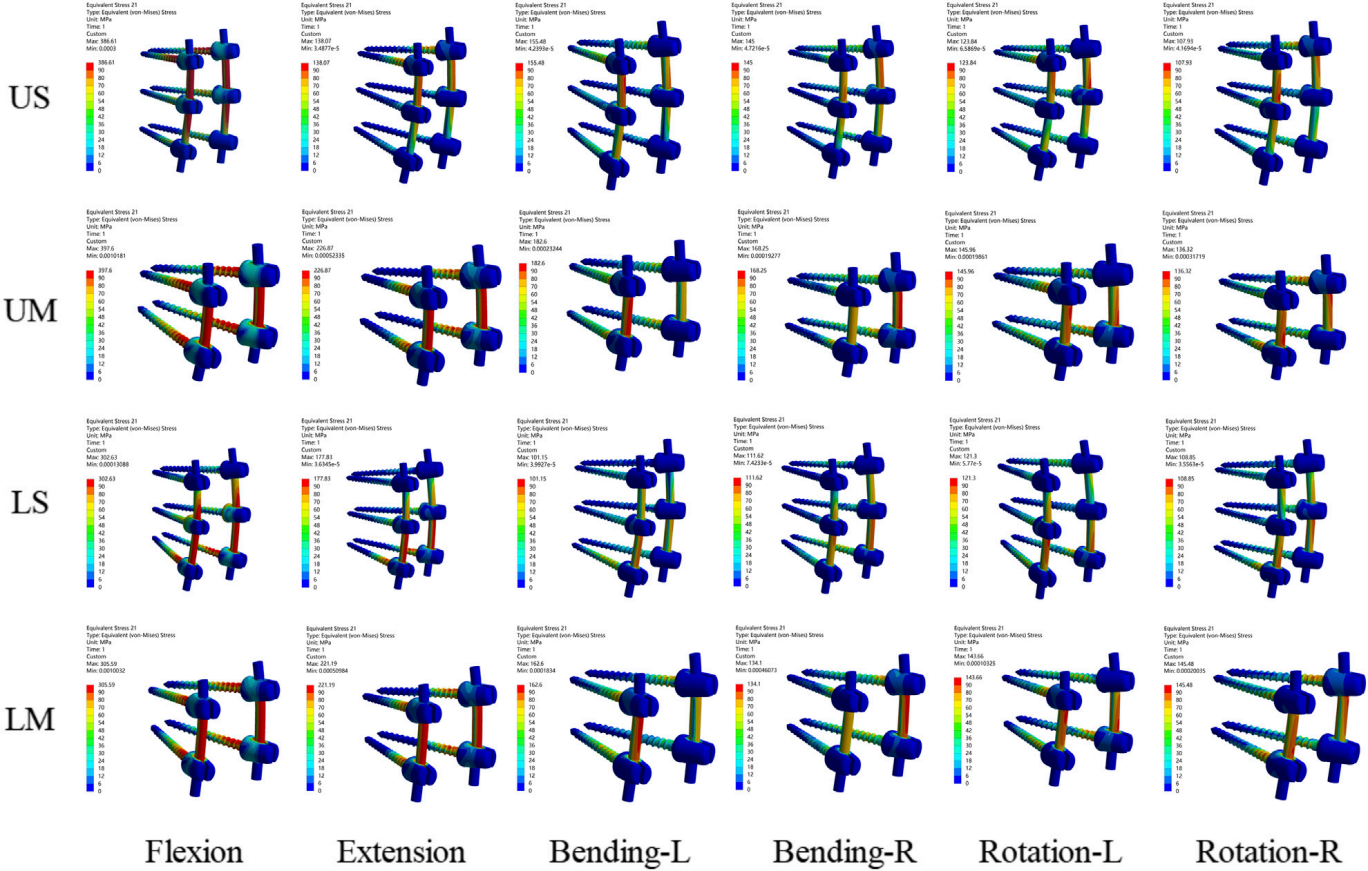
Von Mises stress distribution nephograms of the screw-rod system under six conditions (flexion, extension, left lateral bending, right lateral bending, left rotation, and right rotation) for the four fixation methods. The regions with the highest stress are located at the root of the pedicle screws and the root of the rods.

The peak von Mises stress of proximal screws in the UM group was 15.46% higher than that of distal screws. In the LM group, the peak von Mises stress of distal screws was 13.03% higher than that of proximal screws. In the US group, the peak von Mises stress of proximal screws was 36.22% higher than that of middle screws and 361.57% higher than that of distal screws. In the LS group, the peak von Mises stress of distal screws was 26.12% higher than that of middle screws and 402.87% higher than that of proximal screws. The von Mises stress and stress distribution of the screw-rod system are shown in Figures 6, 7.

#### 3.1.8 Von mises stress of the injured T12 vertebra

**FIGURE 8:**
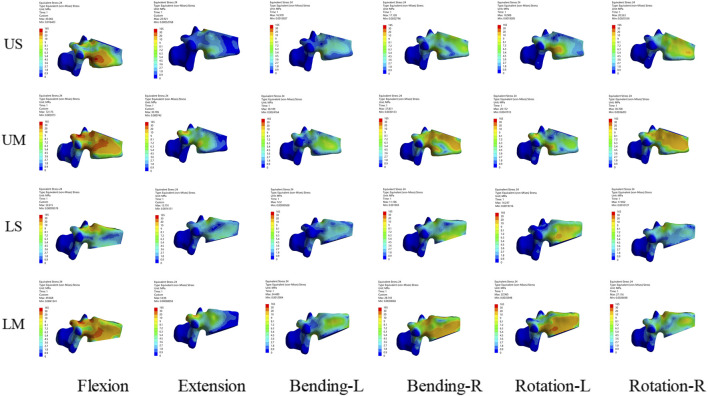
Stress nephograms of the injured T12 vertebra for the four fixation methods show the stress under six conditions including flexion, extension, left lateral bending, right lateral bending, left rotation, and right rotation. The stress of the injured vertebra is mainly concentrated in the middle column and posterior column, while for mono-segment fixation, the stress extends further to the anterior column.

The peak von Mises stress of the injured T12 vertebra in each group occurred under flexion. The peak von Mises stress values of the injured T12 vertebra in the US, UM, LS, and LM groups were 43.066 Mpa, 72.175 Mpa, 32.615 Mpa, and 49.668 Mpa, respectively. After all fixation procedures, the von Mises stress nephograms were concentrated in the middle and posterior columns of the injured vertebra, particularly at the junction of the pedicles and the vertebral body. For single-segment fixation, the von Mises stress range of the injured vertebra extended further to the anterior column. The stress nephogram of the injured T12 vertebra is shown in Figure 8.

### 3.2 Clinical follow-up

#### 3.2.1 Perioperative performance

For the two groups, the chi-square test and Fisher’s exact test were applied to analyze categorical variables including fracture type, segment, gender, cause of injury, and incision healing grade. The independent samples t-test was used to compare continuous variables such as age, operation time, incision length, time to weight-bearing time, hospital stay, Body Mass Index (BMI), and time from injury to operation. The Mann-Whitney U test was adopted for analyzing blood loss and drainage volume. Details of the perioperative conditions are presented in Table 4.

**TABLE 4:** Baseline data and perioperative performance of group 1 (single-segment fixation) and group 2 (short-segment fixation).

Parameter	Group 1 (N = 38)	Group 2 (N = 39)	χ2/t/z	P
① Fracture type			0.187	1.000
Fracture-lower	2 (5.26%)	3 (7.69%)		
Fracture-upper	36 (94.74%)	36 (92.31%)		
② Segment			2.245	0.523
T11	6 (15.79%)	7 (17.95%)		
T12	11 (28.95%)	12 (30.77%)		
L1	10 (26.32%)	14 (35.90%)		
L2	11 (28.95%)	6 (15.38%)		
③ Gender			0.667	0.414
Female	23 (60.53%)	20 (51.28%)		
Male	15 (39.47%)	19 (48.72%)		
④ Injury cause			0.219	0.896
Height falling	13 (34.21%)	13 (33.33%)		
Traffic accident	9 (23.68%)	11 (28.21%)		
Slip	16 (42.11%)	15 (38.46%)		
⑤ Wound healing grade			0.987	1.000
GrageⅠ	37 (97.37%)	37 (94.87%)		
GradeⅡ	1 (2.63%)	1 (2.56%)		
GradeⅢ	0 (0.00%)	1 (2.56%)		
⑥ Age(y)	45.50 ± 15.42	44.87 ± 13.51	0.190	0.850
⑦ Surgery time (h)	100.55 ± 34.40	121.05 ± 35.98	−2.554	0.013
⑧ Incision length (cm)	7.37 ± 1.48	9.44 ± 1.52	−6.053	<0.001
⑨ Blood loss (ml)	140.00 [110.00; 160.00]	270.00 [165.00; 340.00]	−5.466	<0.001
⑩ Drainage volume (ml)	127.50 [105.00; 153.75]	230.00 [182.50; 260.00]	−6.351	<0.001
⑪ Weight-bearing time(d)	2.08 ± 0.91	2.87 ± 1.17	−3.304	0.001
⑫ Hospital time (d)	6.97 ± 1.98	8.15 ± 2.50	−2.294	0.025
⑬ BMI	27.28 ± 4.19	26.10 ± 4.69	1.166	0.247
⑭ Time from injury to surgery(d)	3.97 ± 2.12	4.10 ± 2.07	−0.269	0.788
⑮ Internal fixation device removal time (mo)	18.66 ± 5.15	19.13 ± 4.65	−0.421	0.675

y, year; h, hour; cm, centimeter; ml, milliliter; d, day; mo, month; group1, mono-segment; group2, short-segement; χ², chi-square test; t, t-test; z,z-test; P, Probability value.

The results showed that the operation time, incision length, blood loss, drainage volume, time to weight-bearing time, and hospital stay in Group 2 were significantly higher than those in Group 1 (*P <* 0.05), while there were no significant differences in the other indicators between the two groups (*P >* 0.05). It is worth noting that due to the low incidence of lower endplate fractures, the vast majority of patients in the clinical outcomes of this study had upper endplate fractures; therefore, the prediction of clinical outcomes mainly focuses on the type of upper endplate fractures.

#### 3.2.2 Clinical functional and radiological outcomes

**FIGURE 9:**
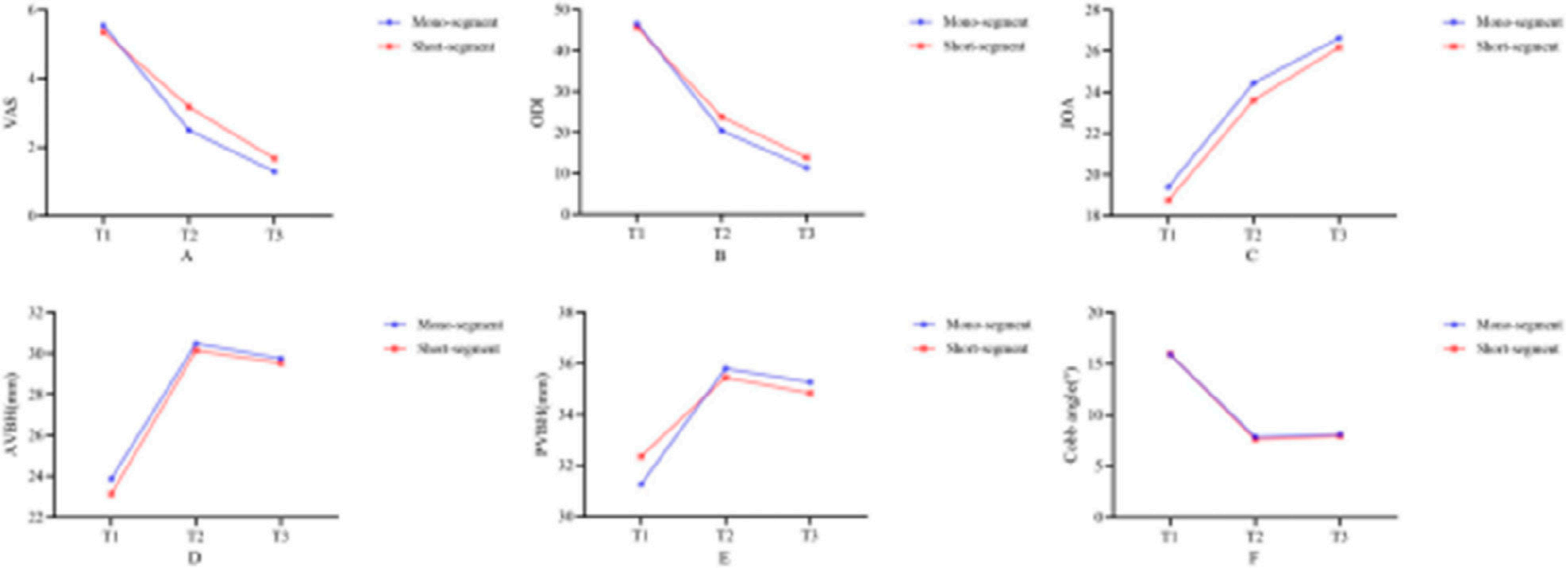
Line charts showing changes in clinical functional indicators and radiological indicators of Group 1 (single-segment fixation) and Group 2 (short-segment fixation) before surgery, immediately after surgery, and at long-term follow-up. **(A)** represents VAS, **(B)** represents ODI, **(C)** represents JOA, **(D)** represents AVBH, **(E)** represents PVBH, and **(F)** represents Cobb angle. The values and change trends of each indicator in the two groups are similar.

**FIGURE 10:**
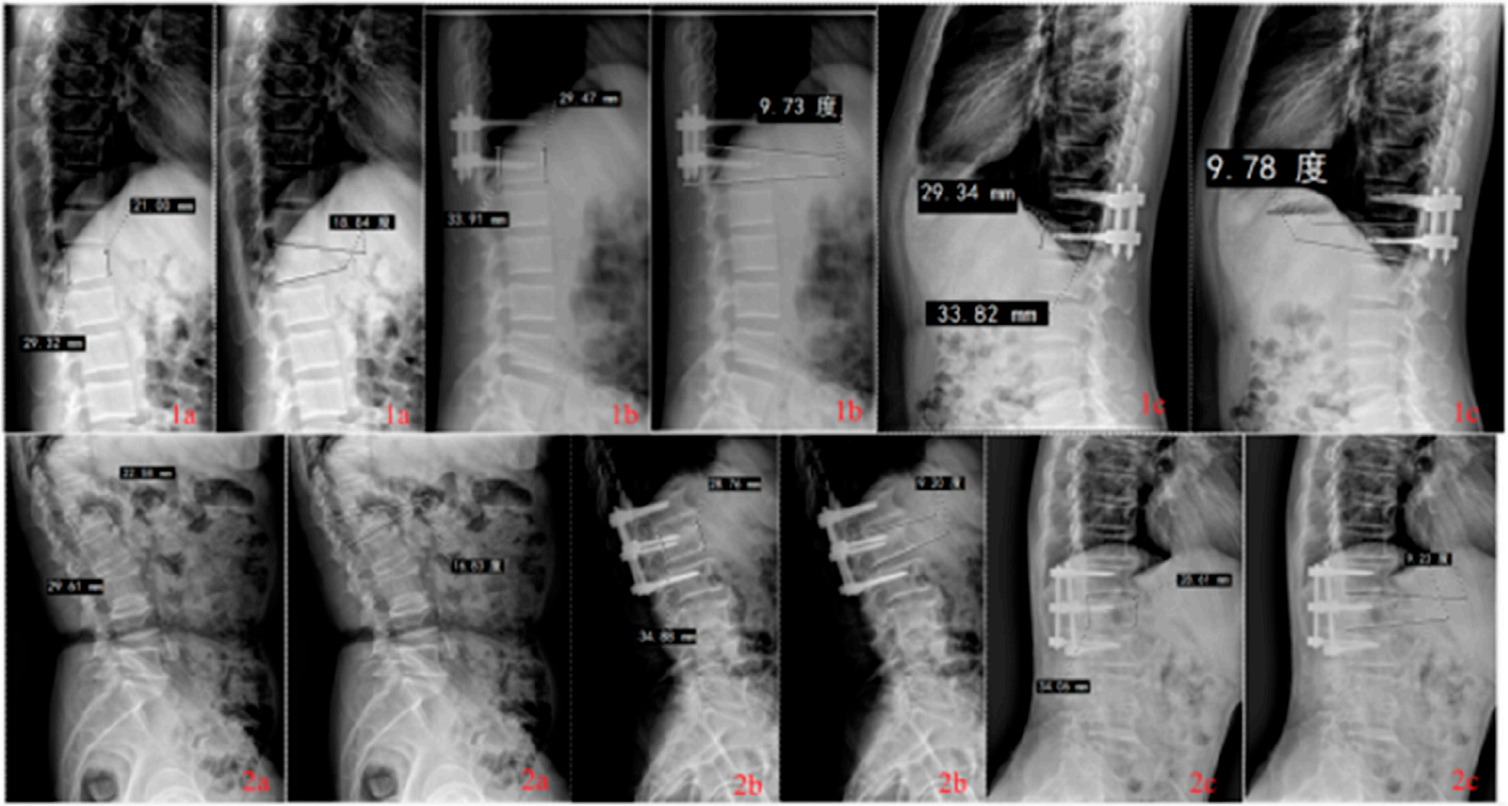
Radiological findings of thoracolumbar fractures in Group 1 (single-segment fixation) and Group 2 (short-segment fixation) before surgery, immediately after surgery, and at long-term follow-up.

A repeated-measures ANOVA with Bonferroni *post hoc* test were performed on VAS, ODI, JOA, AVBH, PVBH, and Cobb angle at different time points between the two groups. The VAS, ODI, JOA, AVBH, PVBH, and cobb angle results of the two groups are shown in Table 5; Figure 9, 10.

**TABLE 5:** Clinical functional indicators (VAS, ODI, JOA) and radiological indicators (AVBH, PVBH, and Cobb angle) of group 1 (single-segment fixation) and group 2 (short-segment fixation) before surgery, immediately after surgery, and at long-term follow-up.

Parameter		T1	T2	T3	P Grouping	P Time	P interaction
VAS	group1(N = 38)	5.55 ± 1.72	2.50 ± 0.95^a^	1.29 ± 1.11^a^ ^b^	0.112	<0.001	0.176
	group2(N = 39)	5.36 ± 1.68	3.18 ± 1.32^a^	1.67 ± 0.93^a^ ^,^ ^b^			
	F	0.250	6.716	2.617			
	P	0.618	0.011	0.110			
ODI	group1(N = 38)	46.66 ± 8.70	20.45 ± 6.25^a^	11.34 ± 5.28^a^ ^b^	0.065	<0.001	0.222
	group29 (N = 39)	45.69 ± 8.31	23.79 ± 7.05^a^	13.85 ± 6.30^a^ ^,^ ^b^			
	F	0.248	4.855	3.564			
	P	0.620	0.031	0.063			
JOA	group1(N = 38)	19.39 ± 2.99	24.45 ± 3.06^a^	26.63 ± 2.02^a^ ^,^ ^b^	0.043	<0.001	0.976
	group2(N = 39)	18.74 ± 2.57	23.62 ± 2.87^a^	25.92 ± 1.97^a^ ^,^ ^b^			
	F	1.051	1.512	2.435			
	P	0.309	0.223	0.123			
AVBH(mm)	group1(N = 38)	23.87 ± 3.22	30.49 ± 3.44^a^	29.76 ± 3.47^a^ ^,^ ^b^	0.433	<0.001	0.556
	group2(N = 39)	23.13 ± 1.16	30.16 ± 2.14^a^	29.53 ± 1.96^a^ ^,^ ^b^			
	F	1.835	0.257	0.121			
	P	0.180	0.613	0.729			
PVBH(mm)	group1(N = 38)	31.26 ± 3.07	35.80 ± 2.80^a^	35.27 ± 2.93^a^ ^,^ ^b^	0.857	<0.001	0.01
	group2(N = 39)	32.35 ± 2.83	35.46 ± 2.47^a^	34.83 ± 2.25^a^ ^,^ ^b^			
	F	2.652	0.334	0.538			
	P	0.108	0.565	0.465			
Cobb angel (°)	group1(N = 38)	15.93 ± 2.47	7.85 ± 1.99^a^	8.10 ± 1.93^a^ ^,^ ^b^	0.755	<0.001	0.452
	group2(N = 39)	15.97 ± 2.75	7.62 ± 1.60^a^	7.95 ± 1.50^a^ ^,^ ^b^			
	F	0.003	0.325	0.156			
	P	0.956	0.570	0.694			

^a^ Compared with the T1 P < 0.05.

^b^ Compared with the T2 P < 0.05; group1,mono-segment; group2,short-segment; T1,pre-operative; T2,immediate post-operative; T3,Long-term follow-up; F,F-text; P,Probability value.

The results showed no interaction between group and time for the VAS, ODI and JOA score (P = 0.176). Inter-group comparison results indicated that there were no significant differences in VAS and ODI scores between the two groups before surgery and at long-term follow-up (*P >* 0.05), while immediately after surgery, the scores of Group 1 were significantly lower than those of Group 2 (*P <* 0.05). No significant differences in JOA scores were observed between the two groups before surgery, immediately after surgery, or at long-term follow-up (*P >* 0.05). Results of intra-group Bonferroni multiple comparisons for both Group 1 and Group 2 revealed that: within each group, VAS and ODI scores immediately after surgery were significantly lower than those before surgery (*P <* 0.05), and scores at long-term follow-up were significantly lower than those both before surgery and immediately after surgery (*P <* 0.05); for JOA scores, values immediately after surgery were significantly higher than those before surgery (*P <* 0.05), and scores at long-term follow-up were significantly higher than those both before surgery and immediately after surgery (*P <* 0.05).

The results showed no interaction between group and time for AVBH or Cobb angle (*P =* 0.556), but a significant interaction between group and time was observed for PVBH (*P =* 0.010). Inter-group comparisons demonstrated no significant differences in AVBH, PVBH, or Cobb angle between the two groups before surgery, immediately after surgery, or at long-term follow-up (*P >* 0.05). Intra-group Bonferroni multiple comparisons for Group 1 and Group 2 showed that: within each group, AVBH and PVBH immediately after surgery were significantly greater than those before surgery (*P <* 0.05), and heights at long-term follow-up were significantly greater than those before surgery but smaller than those immediately after surgery (*P <* 0.05); for Cobb angle, values immediately after surgery were significantly smaller than those before surgery (*P <* 0.05), and angles at long-term follow-up were significantly smaller than those before surgery but larger than those immediately after surgery (*P <* 0.05).

## 4 Discussion

For thoracolumbar burst fractures accompanied by spinal cord or nerve compression, spinal instability, or kyphotic deformity, surgical treatment is often required, and posterior screw-rod system fixation is an effective approach. However, guidelines for surgical methods remain controversial. Existing studies suggest that fixation through the fractured vertebra can improve the stability of the injured vertebra (Cabrera et al., 2022; Suer et al., 2024). The common number of screws used is 4 or 6, and more screws are only considered for extremely severe fractures. For the vast majority of fractures, indirect decompression is performed instead of direct decompression, and both 4 pedicle screw mono-segment fixation and 6 pedicle screw short-segment fixation can achieve indirect decompression (Feng et al., 2024). Spinal fusion is generally not performed for spinal fractures (Lan et al., 2017). The posterior 4 pedicle screw mono-segment surgery offers advantages such as minimal trauma, fewer complications, rapid recovery, and lower surgical difficulty and risk, while effectively restoring spinal height (Wang et al., 2012). Since the upper endplate is parallel to the pedicles and closer to the implanted screws and may thus involve the fractured site, whereas the lower endplate is located below the pedicles and farther from the implanted screws so the screws are therefore almost placed in normal bone tissue, this study further classified thoracolumbar burst fractures into A3.1-type upper endplate fractures and lower endplate fractures to investigate the effects of this anatomical difference. Additionally, endplate reduction has a significant impact on intervertebral disc degeneration (Su et al., 2023). Therefore, this study aimed to evaluate the efficacy of different fixation methods and the influence of anatomical differences in fracture types on treatment and prognosis through finite element analysis of 4 pedicle screw mono-segment trans-injured vertebra fixation and 6 pedicle screw short-segment trans-injured vertebra fixation for superior or lower endplate fractures of the thoracolumbar spine. Ultimately, the study subjects were categorized into four groups: US, UM, LS and LM. The outcome indicators observed included: the ROM of all segments, the von Mises stress of adjacent intervertebral discs, the von Mises stress of adjacent facet joints, the maximum displacement of the fixed segment, the ROM of the fixed segment, the ROM of the injured T12 vertebra, the stress of the injured T12 vertebra, and the stress of pedicle screws and rods.

Anatomy has long been regarded as the cornerstone of medical and related health education, and a good understanding of it enables the safe and effective treatment of numerous diseases affecting the human body. With the advancement of technology, cadavers are no longer the sole tool for anatomical research; computer-based methods are playing an increasingly important role. These include traditional FEA, Probabilistic Finite Element Method (PFEM), Boundary Element Method (BEM), artificial intelligence approaches, and machine-assisted technologies. The shared advantages of these new techniques lie in easy access to materials and simple operation, enabling repeatable research, reducing waste, and greatly improving research efficiency (Santillo et al., 2016; Zhang and Fu, 2024). In this study, finite element method was used to calculate and analyze relevant data. First, 3D reconstructed CT images of the thoracolumbar spine from a healthy, young male without fractures were collected. These images were then imported into Mimics software to extract the 3D bony contours, which were subsequently solidified using Geomagic software. SolidWorks software was employed to construct an intervertebral disc model and two types of internal fixation models. Two types of fracture models were established via V-shaped osteotomy, and all models were assembled. Ansys Workbench software was used to create ligaments, assign material properties, generate meshes, and perform calculation and solution. The lower endplate of L2 was constrained, and a vertical load of 150 N and a torque of 10 Nm were applied to the upper endplate of T10 to measure the ROM in flexion, extension, left lateral bending, right lateral bending, left rotation, and right rotation. The results were consistent with the models reported in previous reference literatures. Referring to the standing posture and weight-bearing status of a volunteer (height: 170 cm, weight: 65 kg) during rehabilitation, an experimental load of 400 N vertical force and 7.5 Nm torque was adopted in this study.

Finite Element Analysis and Prediction of Specific Models, In the same type of fracture, the finite element analysis and prediction of specific models showed that the ROM in all directions of the 4 pedicle screw mono-segment fixation was greater than that of the 6 pedicle screw short-segment fixation. Specifically, the 4 pedicle screw mono-segment fixation better preserved the spinal ROM in all directions. This is particularly relevant for special populations such as athletes, who need to resume functional exercises early to maintain their competitive state and thus have higher requirements for spinal mobility. The von Mises stress on the adjacent intervertebral discs in the 4 pedicle screw mono-segment fixation group was lower than that in the 6 pedicle screw short-segment fixation group, indicating a lower risk of adjacent disc degeneration in the 4 pedicle screw mono-segment fixation. The peak stress of the intervertebral disc was concentrated in the posterior part of the annulus fibrosus; such stress concentration implies an increased likelihood of tearing or rupture of the annulus fibrosus. Similar to the von Mises stress on adjacent discs, the 4 pedicle screw mono-segment fixation also resulted in a lower risk of degeneration of adjacent facet joints. Collectively, the von Mises stress data of adjacent intervertebral discs and facet joints suggest that the 4 pedicle screw mono-segment fixation can reduce the risk of adjacent segment degeneration, which is consistent with the findings of previous studies (Zhang et al., 2024).

The maximum displacement of the fixed segment in 6 pedicle screw short-segment fixation was smaller than that in 4 pedicle screw mono-segment fixation, indicating better stability of the 6 pedicle screw short-segment fixation. The ROM of the fixed segment and the ROM of the injured T12 vertebra also showed similar results to the maximum displacement of the fixed segment, further confirming the stability advantage of the 6 pedicle screw short-segment fixation, which is consistent with previous studies (Mahar et al., 2007; Alizadeh et al., 2013). Therefore, for spinal fractures with severe instability, the 4 pedicle screw mono-segment may not achieve satisfactory efficacy. Currently, a relatively reliable criterion for evaluating the severity of thoracolumbar fractures is the LSC score (Sun et al., 2016). Generally, a score of ≤3-4 points is classified as a mild fracture, while a score of >7 points indicates a severe fracture. This scoring system assesses thoracolumbar fractures based on three dimensions: the degree of vertebral body compression, the degree of comminution, and the degree of kyphosis. It is currently the most effective classification system for reflecting the stability of thoracolumbar fractures and provides an intuitive quantitative assessment of fracture severity.

The peak stress of the rods was higher than that of the screws, and the maximum stress of the screws was concentrated at the screw-rod junction. The peak stresses of the screw-rod system in both 4 pedicle screw mono-segment fixation and 6 pedicle screw short-segment fixation were within the fracture limit of titanium alloy (529 Mpa) (Wycisk et al., 2014). Consistent with the findings on screw-rod system, 6 pedicle screw short-segment fixation was more effective in distributing the stress of the injured vertebra. After all fixation procedures, the von Mises stress nephograms were concentrated in the middle and posterior columns of the injured vertebra, particularly at the junction of the pedicles and the vertebral body. In contrast, the von Mises stress range of the injured vertebra in mono-segment fixation extended further to the anterior column. Therefore, for special patients with poor bone mineral density (e.g., osteoporosis), 4 pedicle screw mono-segment fixation may pose a higher risk of screw loosening and anterior column height loss. Of course, for populations with normal bone mineral density and good bone quality, the stronger load-bearing capacity at the screw-bone interface significantly reduces the risk of screw loosening (Akabane et al., 2025). Currently, it is recommended that patients with osteoporotic thoracolumbar fractures be treated in accordance with professional guidelines, such as the DGOU-OF classification proposed by AO Spine. This classification is based on vertebral deformation; a score greater than 6 typically indicates the need for surgical intervention, and the surgical approach varies by fracture type. For example, if vertebral integrity is lost, posterior internal fixation combined with bone cement augmentation of the fractured vertebra or long-segment posterior internal fixation may be selected; for vertebral collapse, long-segment posterior internal fixation is an option. If there is neurological deficit, posterior decompression is recommended on the basis of fixation/fusion. Corresponding fixation recommendations are also provided for special cases such as multi-segment fractures. In addition, Zhang et al. (Zhang et al., 2023) compared the efficacy of cement-augmented pedicle screw fixation (CAPS), long-segment pedicle screw fixation (LSPS), cortical bone trajectory (CBT) screw fixation, and combined CBT and pedicle screw fixation (CBT-PS) in the treatment of osteoporotic thoracolumbar burst fractures. The results indicated that CAPS and CBT-PS might be superior options. This suggests that for patients with mild osteoporotic thoracolumbar fractures, the use of 4 cement-augmented screws or single-segment CBT-PS may also help enhance the load-bearing capacity of the screw-bone interface. Furthermore, compared with 4 pedicle screw mono-segment fixation, longer-segment fixation carries a higher risk of fatigue fracture (Benson et al., 1992; Soshi et al., 1991; Wittenberg et al., 1991). Wolff’s Law states that vertical forces along the fracture line can promote bone healing and continuous thickening of the callus. Excessive fixation techniques that hinder bone growth are not recommended, which highlights the positive role of stress on the injured vertebra (Ma et al., 2023).

In the same fixation type, the ROM of all directions was greater for lower endplate fractures than for upper endplate fractures. Lower endplate fractures better preserved the ROM of the spine in all directions. The von Mises stress of the adjacent intervertebral discs in upper endplate fractures was greater than that in lower endplate fractures, indicating a lower risk of adjacent intervertebral disc degeneration in lower endplate fractures. The peak von Mises stress of the adjacent intervertebral discs in upper endplate fractures occurred at the proximal end, while the peak von Mises stress in lower endplate fractures occurred at the distal end. Similar to the von Mises stress of the adjacent intervertebral discs, the risk of adjacent facet joint degeneration was lower in lower endplate fractures. The peak von Mises stress of the adjacent facet joints in upper endplate fractures also occurred at the proximal end, while the peak von Mises stress in lower endplate fractures occurred at the distal end. The von Mises stress of the adjacent intervertebral discs and facet joints suggested that lower endplate fractures had a lower risk of adjacent segment degeneration compared to upper endplate fractures. The proximal adjacent segment may be the stress concentration area in upper endplate fractures, while the distal adjacent segment may be the stress concentration area in lower endplate fractures. The maximum displacement of the fixed segment in lower endplate fractures was lower than that in upper endplate fractures, indicating better stability in lower endplate fractures. The ROM of the fixed segment and the ROM of the injured vertebra T12 were also similar to the results of the maximum displacement of the fixed segment, further indicating the stability advantage of lower endplate fractures. Moreover, the average instability ratio indicated that the stability gap between 4 pedicle screw mono-segment and 6 pedicle screw short-segment fixations was smaller for lower endplate fractures than for upper endplate fractures. The von Mises stress of the screws and rods in lower endplate fractures was lower than that in upper endplate fractures, indicating better stress distribution in lower endplate fractures. The maximum screw stress occurred at the proximal end in upper endplate fractures, while the maximum screw stress occurred at the distal end in lower endplate fractures, which may suggest the use of two additional coarse screws (Lin et al., 2016). Similar to the results of the screws and rods, lower endplate fractures could better distribute the stress on the injured vertebra.

During clinical follow-up, Perioperative performance: The 4 pedicle screw mono-segment group showed superior outcomes in operative time [(100.55 ± 34.40) min vs. (121.05 ± 35.98) min, *P <* 0.05], incision length [(7.37 ± 1.48) cm vs. (9.44 ± 1.52) cm, *P <* 0.05], blood loss [(140.00 [110.00; 160.00]) mL vs. (270.00 [165.00; 340.00]) mL, *P <* 0.05], drainage volume [(127.50 [105.00; 153.75]) mL vs. (230.00 [182.50; 260.00]) mL, *P <* 0.05], Weight-bearing time [(2.08 ± 0.91) d vs. (2.87 ± 1.17) d, *P <* 0.05], and hospital time [(6.97 ± 1.98) d vs. (8.15 ± 2.50) d, P < 0.05] compared with the 6 pedicle screw short-segment fixation group. Clinical Functional performance: Immediate post-operative, VAS and ODI scores in the 4 pedicle screw group were significantly lower than those in the 6 pedicle screw group (P < 0.05), while there were no significant differences in VAS, ODI, and JOA between the two groups for other time points (*P >* 0.05). All three scores showed significant improvement over time (P < 0.05). Imaging performance: No significant differences were observed in AVBH, PVBH, or cobb angle between the two groups at pre-operative, immediate post-operative, and long-term follow-up time points (P > 0.05). Within both groups, the AVBH, PVBH, and cobb angle were significantly better at immediate post-operative and long-term follow-up than pre-operatively (P < 0.05), but correction at long-term follow-up was lost compared with the immediate post-operative period (P < 0.05). Although finite element analysis suggests that 6 pedicle screw short-segment fixation provides better stability, during the fracture healing period from surgery to internal fixation removal, 4 pedicle screw mono-segment fixation can provide sufficient short-term stability for mild to moderate fractures to promote morphological healing of the injured vertebra.

### 4.1 Limitations

① This model was based solely on data from a single healthy volunteer. Due to limitations imposed by individual anatomical characteristics (e.g., age, body weight, BMD), future studies should expand the research scope to include patient models with diverse anatomical features or data from a larger population. Alternatively, the PFEM could be employed to capture inter-individual variability, thereby achieving more reliable population-level biomechanical predictions. ② The model excluded certain soft tissues, such as muscles or spinal nerves. Future studies should consider expanding the model to include, at minimum, a simplified representation of muscles that influence force distribution and spinal segment stability (Sun et al., 2019)—for instance, using ligament-like rod elements. This modification would enable a more realistic simulation of spinal biomechanical characteristics. ③ Given that the mechanical modeling logic of finite element studies and the statistical data analysis logic of statistics differ in focus—the former emphasizes “mechanical behavior of a specific model,” while the latter focuses on “patterns in population data”—statistical methods serve as auxiliary tools rather than core methods in spinal finite element analysis. Consequently, statistical analysis of inter-model differences was not incorporated in this finite element study. However, future research should integrate statistical significance analysis of inter-model differences (e.g., ANOVA, t-tests) to enhance the reliability of comparisons between models. ④ Mechanical properties were not included in this study, future research requires further investigation through cadaveric biomechanical studies.⑤ For 4 pedicle screw mono-segment fixation in upper endplate fractures, the normal reference vertebra was the superior adjacent vertebra of the injured vertebra and is closer to the thoracic spine. In contrast, for 4 pedicle screw mono-segment fixation in lower endplate fractures, the normal reference vertebra was the inferior adjacent vertebra of the injured vertebra and is closer to the lumbar spine. This confounding factor, which combines anatomical differences and fixation method variations, may have simultaneously affected both finite element prediction results and clinical outcomes. Specifically, the observed differences between the two fixation methods might partially stem from this structural asymmetry rather than solely from the fixation technique itself, though this issue is unavoidable.⑥ The clinical follow-up analysis was retrospective, and this study primarily focused on patients with upper endplate fractures. Due to the limited number of cases involving lower endplate fractures, a comparison of reduction efficacy across different fracture types could not be conducted. Future research should adopt prospective, randomized clinical designs and collect more cases of lower endplate fractures to enable comparative analysis of reduction efficacy among different fracture types.⑦ The follow-up duration was 18.66 ± 5.15 months for the mono-segment fixation group and 19.13 ± 4.65 months for the short-segment fixation group. This duration only reflects short-to-medium-term efficacy and cannot fully evaluate the long-term stability and safety of different fixation strategies, which refers to 3–5 years postoperatively. Subsequent studies plan to extend follow-up to 3 years to further validate the long-term efficacy and biomechanical impacts of various fixation strategies.

## 5 Conclusion

1. Finite element analysis and prediction based on specific models: Both 4 pedicle screw mono-segment fixation and 6 pedicle screw short-segment fixation are effective methods for treating thoracolumbar burst fractures of the spine. The 4 pedicle screw mono-segment fixation may be suitable for patients with normal bone mineral density and mild-to-moderate fractures, with advantages of preserving spinal mobility and reducing the risk of adjacent segment degeneration. The 6 pedicle screw short-segment fixation has a wider application range, and its advantages lie in better stability and stress dispersion. For upper endplate fractures, the adjacent segment at the proximal end is the stress concentration area, and the proximal screws bear the maximum stress; in contrast, for lower endplate fractures, the adjacent segment at the distal end is the stress concentration area, and the distal screws bear the maximum stress. The stability gap between 4 pedicle screw mono-segment fixation and 6 pedicle screw short-segment fixation is smaller in lower endplate fractures than in upper endplate fractures. Compared with upper endplate fractures, lower endplate fractures show better overall performance and may have a better prognosis. However, different finite element models may be required in the future to reduce the impact of individual differences.
2. Clinical outcomes mainly for upper endplate fractures: For mild-to-moderate fractures, the 4 pedicle screw mono-segment fixation achieves the same reduction effect as the 6 pedicle screw short-segment fixation both immediately after surgery and during long-term internal fixation removal. Moreover, it has advantages such as shorter operation time, smaller incision, less blood loss, earlier weight-bearing time, shorter hospital stay, less early postoperative pain, and faster functional recovery. However, more cases of lower endplate fractures may need to be collected in the future for further verification.

## Data Availability

The raw data supporting the conclusions of this article will be made available by the authors, without undue reservation.
